# Glucocorticoid-Induced Leucine Zipper: Fine-Tuning of Dendritic Cells Function

**DOI:** 10.3389/fimmu.2018.01232

**Published:** 2018-06-04

**Authors:** Mathias Vétillard, Géraldine Schlecht-Louf

**Affiliations:** UMR996-Inflammation, Chimiokines et Immunopathologie, INSERM, Faculté de médecine, Univ Paris-Sud, Université Paris-Saclay, Clamart, France

**Keywords:** dendritic cells, tolerance, TSC22D3, glucocorticoid-induced leucine zipper, antigen presentation, regulatory T cells

## Abstract

Dendritic cells (DCs) are key antigen-presenting cells that control the induction of both tolerance and immunity. Understanding the molecular mechanisms regulating DCs commitment toward a regulatory- or effector-inducing profile is critical for better designing prophylactic and therapeutic approaches. Initially identified in dexamethasone-treated thymocytes, the glucocorticoid-induced leucine zipper (GILZ) protein has emerged as a critical factor mediating most, but not all, glucocorticoids effects in both non-immune and immune cells. This intracellular protein exerts pleiotropic effects through interactions with transcription factors and signaling proteins, thus modulating signal transduction and gene expression. GILZ has been reported to control the proliferation, survival, and differentiation of lymphocytes, while its expression confers anti-inflammatory phenotype to monocytes and macrophages. In the past twelve years, a growing set of data has also established that GILZ expression in DCs is a molecular switch controlling their T-cell-priming capacity. Here, after a brief presentation of GILZ isoforms and functions, we summarize current knowledge regarding GILZ expression and regulation in DCs, in both health and disease. We further present the functional consequences of GILZ expression on DCs capacity to prime effector or regulatory T-cell responses and highlight recent findings pointing to a broader role of GILZ in the fine tuning of antigen capture, processing, and presentation by DCs. Finally, we discuss future prospects regarding the possible roles for GILZ in the control of DCs function in the steady state and in the context of infections and chronic pathologies.

## Introduction

Dendritic cells (DCs) are the most potent antigen-presenting cells (APCs), with recognized ability to orchestrate tolerance and immunity. In parallel to antigen processing, they integrate antigen- and microenvironment-associated stimuli and translate them into membrane and cytokine signals for appropriate T-cell priming and polarization or T-cell tolerization (1). Thus, DCs are key regulators of immune homeostasis and gaining knowledge in the mechanisms controlling their polarization toward tolerogenic or immunogenic APCs is of critical importance for both prophylactic and therapeutic approaches in allergy, autoimmunity, inflammatory diseases, infections, and cancers.

Transcriptomic, phenotypic, and functional analyses have identified three steady-state DC subsets in human and mouse (2, 3). Conventional DCs (cDCs) excel in antigen presentation and encompass two subpopulations, namely cDC1 and cDC2, the former being functionally specialized in cross-presentation and type 1 T-helper (Th1) responses induction and the latter promoting Th2 and Th17 T-cell responses (4). The third subset corresponds to plasmacytoid DCs (pDCs) that are specialized in antiviral immunity (5). While each DC subset displays specialized immune-activating functions, all of them have also been shown to promote tolerance and favor regulatory T cells (Treg) differentiation, expansion, and/or activation in certain contexts. This points to mechanisms controlling DCs’ functional switch between tolerogenic and immunogenic APCs (6–8). In addition to these three well-defined DC subsets, inflammatory DCs can arise from monocytes in the course of inflammation, which contribute to innate immune responses and T-cell priming (9).

Among factors reported to skew DCs maturation toward a tolerogenic profile (10), glucocorticoids (GCs), rapamycine, interleukin (IL)-10, transforming growth factor-β (TGF-β), and vitamin D3 (vitD3) have been shown to promote the expression of the glucocorticoid-induced leucine zipper (GILZ) protein (11). GILZ was initially described in murine thymocytes treated with synthetic GCs (Dexamethasone, Dex) (12), but this intracellular protein is expressed in most tissues, including immune cells (12–19). GILZ has since been demonstrated to mediate GCs’ effects in human DCs (20, 21) and has more generally emerged as a regulator of DCs tolerogenic function in both mouse and human (20–27). More recently, we have unraveled that GILZ expression by DCs controls their efficiency at antigen capture and cross-presentation (27), suggesting that the extent of GILZ action in DCs may be broader than initially expected.

Herein, we provide a comprehensive review of recent insights into GILZ expression and functions in DCs and emphasize its implication as a regulator of DCs function, which modulates key processes ranging from antigen capture and presentation to functional maturation and T-cell priming.

## Gilz’s General Properties

**Figure 1:**
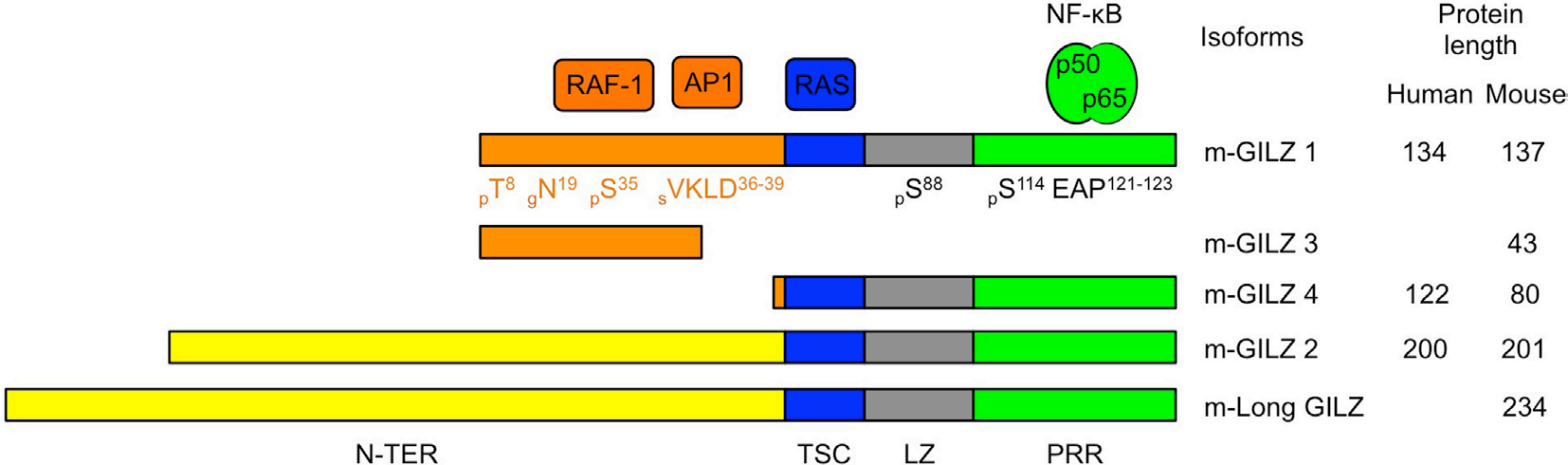
Glucocorticoid-induced leucine zipper (GILZ) isoforms and partners. Five murine GILZ (m-GILZ) isoforms, namely m-GILZ 1–4 and long GILZ (m-Long GILZ) have been identified in mouse (13, 28), with different N-TER domains while m-GILZ1, 2, 4 and m-Long GILZ encompass conserved TSC, LZ, and PRR domains. RAF1 (37) and AP1 (33) interact with GILZ1 and presumably GILZ3 N-TER domain. RAS interacts with the TSC (blue) domain (38). LZ (gray) domain allows dimerization (33). Nuclear factor Kappa B interacts with the PRR (green) domain and requires an EAP motif in positions 121–123 of GILZ1 (39). Predicted posttranslational modification sites are annotated with their positions (^X^) and the nature of the modification, i.e., phosphorylation, p; glycosylation, g; and sumoylation, s. *Tsc22-d3* transcription starts either at a canonical AUG codon or at an upstream non-canonical AUG codon. GILZ isoforms derived from the use of the same codon display identical N-TER domain (orange or yellow, respectively). Protein sequences were aligned using BLAST. The scale is proportional to the real size of the protein. N-TER, N-terminal domain; TSC, TGF-β-stimulated clone; LZ, leucine zipper; PRR, proline-rich region.

Glucocorticoid-induced leucine zipper is encoded by the TGF-β-stimulated clone (TSC) *22 domain family protein 3* (*Tsc22d3*) gene located on the X-chromosome and is constitutively expressed in most tissues (12, 16, 28). GILZ is among the earliest and highest GC-induced genes. In addition to GC response elements, GILZ promoter harbors binding sites for several transcription factors, including Forkhead-Box O3, C-AMP Response Element-Binding protein, and Serum Responsive Factor. Two E-boxes, one GATA Box as well as putative-binding sites for signal transducer and activator of transcription 6, nuclear factor of activated T-cells and Octamer are also reported (29–32). Five GILZ isoforms exist in mouse, generated upon use of alternative initiation and splicing sites (13, 28). They differ in their N-terminal parts but four of them share conserved TSC and Leucine Zipper (LZ) domains and a C-terminal proline-rich region (PRR) (Figure 1). The term GILZ usually refers to the 137 amino acid (aa)-long GILZ1/*Tsc22d3-2* protein in mouse and to the 134 aa-long isoform in human. As only this canonical isoform has been studied in DCs so far (20, 23, 24, 26, 27), we will adopt this nomenclature. Nevertheless, the other GILZ isoforms could be of functional relevance in DCs, as they may modulate GILZ1 function upon dimerization (33), competition, or ensure autonomous function owing to their unique N-terminal domain. GILZ has a short half-life [2–3 h (34–36)] and is quickly degraded upon ubiquitin-proteasome degradation (34). Several sites for posttranslational modification have been predicted in GILZ sequence (Figure 1), but only polyubiquitination by K48 ubiquitins has been confirmed so far (34).

In mouse lymphocytes, GILZ controls a wide range of processes including their activation, proliferation, survival, and differentiation (12, 40–43). GILZ also confers anti-inflammatory phenotype to innate immune cells, including human monocytes (25, 44, 45), mouse and human macrophages (35, 36, 46–48), human mastocytes (49), and mouse and human neutrophils (50, 51). Studies mostly done in T cells have reported that GILZ can exert its pleiotropic effects through direct interactions with transcription factors and signaling proteins, according to its cytoplasmic and nuclear distribution (12). GILZ directly binds to NF-κB-p65 (39, 46, 52), AP-1 (33), C/EBP (53), PU.1 (51) and prevents the nuclear translocation of FoxO3 (54). GILZ also interacts with Ras, Raf-1, and mTORC2, thereby inhibiting the MEK/ERK-1/2 and mTORC2/AKT pathways respectively (37, 38, 55). Finally, GILZ can also associate with nuclear DNA (19, 51, 56, 57) and modulate transcription upon competition with positive Th17-polarization regulators (56, 57) or relief of PU.1-mediated repression (51).

## Gilz Expression in DCs

**Figure 2:**
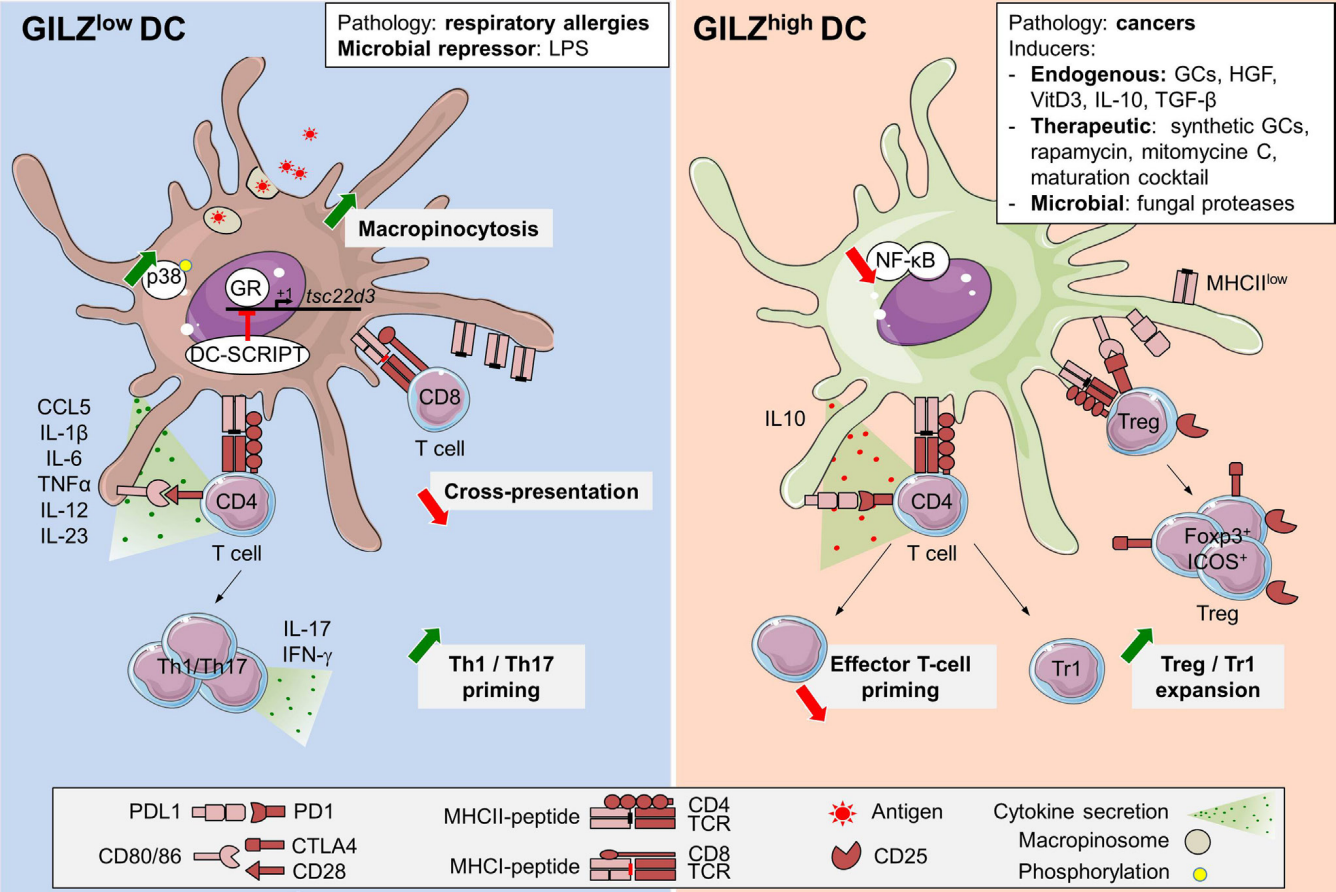
Glucocorticoid-induced leucine zipper (GILZ) regulation and functions in dendritic cells (DCs). GILZ expression in DCs can be induced by steady-state factors (11, 21, 22), immunosuppressive cytokines like TGF-β or interleukin (IL)-10 (20, 25), DC-maturation cocktail containing TNF-α, IL-1β, IL-6, and PGE2 (24), cell-derived factors (26, 45, 67), immunosuppressive drugs as synthetic GCs, rapamycin, and mitomycine C (11), fungal proteases (68), and cancer microenvironment (26). So far, the only exogenous GILZ repressor reported in DCs is LPS (26, 27). In addition, GILZ levels are reduced in blood DCs from respiratory allergic patients (21). DC-SCRIPT acts as an endogenous GR-repressor, thus limiting GILZ induction upon GCs exposure (64, 65). High GILZ levels promote PD-L1 expression and IL-10 production while limiting IL-12 and IL-23 secretion. Thus, GILZ^hi^ DCs are poor inducers of Th1 and Th17 T cells but efficient Treg and Tr1 activators (20, 23, 25). GILZ inhibits NF-κB functions upon interaction with p65 subunit (39, 46, 52). GILZ repression promotes macropinocytosis, likely upon increased p38-MAPK phosphorylation (27). GILZ deletion reduces antigen cross-presentation (27). GCs, glucocorticoids; TGF-β, transforming growth factor-β; IL-10, interleukin-10; HGF, hepatocyte growth factor; LPS, lipopolysaccharide; ICOS, inducible co-stimulator; Foxp3, Forkhead box p3; NF-κB, nuclear factor kappa B; CTLA4, cytotoxic T-lymphocyte-associated protein; Tr1, Type 1 regulatory T cells; Treg, regulatory T cells; p38-MAPK, p38 mitogen-activated protein kinase; GR, glucocorticoid receptor; cDC, conventional dendritic cell; *tsc22d3*, TGF-β-stimulated clone (TSC) *22 domain family protein 3*.

Glucocorticoid-induced leucine zipper expression by steady-state human and murine DCs was initially reported by our group, using quantitative RT-PCR, Western Blot, and flow cytometry (21, 23, 27). In mouse, we further documented heterogeneity in GILZ levels among splenic DC subsets, the cDC1 population displaying higher levels than the cDC2 and pDC subsets (27). As in monocytes (25, 44, 45), GILZ mRNA is quickly downregulated upon human blood DCs culture *ex vivo* (21), pointing to the requirement for an active mechanism to maintain GILZ expression in these cells. This tonic signal is most likely provided, at least in part, by endogenous GCs as *in vivo* glucocorticoid receptor (GR) blockade reduces GILZ expression in murine splenocytes (58). Accordingly, GILZ mRNA levels follow robust circadian rhythm in adipose tissue and muscle (59–61), and increase, upon restraint stress, in spleen and macrophages (62). However, daytime and stress-induced variations of GILZ levels in DCs have not been explored so far. As in other cell types, exogenous GCs promote GILZ expression in DCs, both *in vitro* and *in vivo* (11, 20, 21, 25, 58, 63). Thus, GILZ expression, which is quite low in mouse bone marrow-derived DCs (BMDCs) (23, 26, 27) and absent in human monocytes-derived DCs (Mo-DCs) (20, 45) is dose-dependently upregulated upon Dex treatment (11, 20, 45). GILZ transcription in Mo-DCs is modulated by DC-SCRIPT, a co-repressor of GR (64, 65) (Figure 2). Remarkably, DC-SCRIPT mRNA is selectively and highly expressed in cDCs in the steady state, but not in other immune cells [The immunological Genome Project (66)], suggesting a cDC-specific limitation of GILZ induction by GCs. While the contribution of DC-SCRIPT to GILZ level control in DC-subsets and its modulation upon cell-activation remain to be explored *in vivo*, the selective limitation of GR-induced GILZ expression in cDCs could potentiate the impact of other GILZ-inducing factors in these cells. Indeed, several molecules present in tissues at the steady state, such as vitD3 (11) and hepatocyte growth factor (HGF) (22), or produced in immunosuppressive microenvironments, as IL-10 and TGF-β, promote GILZ expression in DCs (20, 22, 25), supporting the hypothesis that GILZ is primarily induced in anti-inflammatory contexts (Figure 2). However, GILZ is also overexpressed in clinical grade Mo-DCs exposed to a maturation cocktail containing TNF-α, IL-1β, IL-6, and PGE2 (24), suggesting that pro-inflammatory factors can upregulate GILZ levels in certain conditions.

So far, few microbial products were tested for their ability to control GILZ expression in DCs. Mouse DCs exposure to lipopolysaccharides downregulates GILZ at the transcript and protein levels, *in vitro* and *in vivo* (26, 27) (Figure 2). Conversely, fungal proteases from *Aspergillus oryzae* promote GILZ overexpression in human Mo-DCs, independently from the GR (68). Alteration of GILZ expression by microbial products has been reported in other cell types. In human epithelial cells, *Yersinia enterocolitica* YopT and *Clostridium difficile* Toxin B induce GILZ expression through USF-1 and -2 binding to *TSC22D3* promoter (31). In microglia, *Tsc22d3* was among the most downregulated genes from antibiotic-treated mice, suggesting a possible contribution of tonic signals from microbiota to GILZ levels control (69). Regarding viruses, Chikungunya and Respiratory Syncytial Virus infections downregulate GILZ expression in primary murine astrocytes (70) and in human epithelial cells (71), respectively. Conversely, Infectious Bursal Disease Virus protein VP4 suppresses GILZ degradation (34), thereby preventing type 1 IFN production in human fibroblast and keratinocyte cell lines (72). Whether GILZ modulation in the course of infections also occurs in DCs and the possible consequences and importance of such regulation remain to be addressed.

Altered GILZ expression in DCs has also been reported in the course of chronic pathologies. Blood DCs from respiratory allergic patients harbor reduced GILZ levels as compared to non-allergic healthy volunteers (21). Besides, GILZ overexpression has been found in tumor-associated DCs from A20 B-cell lymphoma-engrafted mice (26). While GILZ levels in tumor-infiltratring DCs have not been studied in humans so far, the hypothesis that GILZ could be induced in such pathologies is supported by the high GILZ expression detected in infiltrating macrophages in Burkitt’s lymphoma (46). Regarding the mechanisms that could contribute to such GILZ induction, Wang et al. found that hypoxia upregulated GILZ expression in macrophages and rats spleens (62). In addition, Lebson et al. reported GILZ induction in BMDCs exposed to A20- and B16-tumor cells conditioned medium, pointing to a role for soluble factors in this increased expression (26). These factors may include known GILZ inducers as reported in epithelial cell-conditioned medium-treated BMDCs (67). Additional studies have pointed to GILZ levels modulation in cells other than DCs during chronic inflammation. Thus, GILZ expression is reduced in skin lesions from atopic dermatitis (73) and psoriasis patients (63), as well as in macrophages from Crohn’s disease granuloma (46). Conversely, GILZ is overexpressed in the synovia of patients with active rheumatoid arthritis (RA) (58). Whether GILZ acts as an endogenous inhibitor of inflammation, as suggested by exacerbated imiquimod-induced psoriatic-like lesions in GILZ knock-out mice (63) and increased inflammation upon GILZ knock-down in synovial tissues in a murine model of RA (58), or whether it primarily mediates exogenous GCs effects (43) remains debated. The contribution of GILZ induction to GCs therapeutic effects is supported by several studies, two of them having assessed it in DCs. First, restoration of GILZ expression in DCs to normal levels was required for the increase of IL-10^+^CD4^+^ T cells, known to mediate oral GC-therapy beneficial effects in respiratory allergic patients (21). Second, in lupus patients, GILZ/prednisolone ratios in pDCs and myeloid DCs exhibited a negative correlation with disease activity (74). In addition to GCs, several therapeutic agents have been reported to induce GILZ in DCs, although the importance of this expression for their effects has not been explored. Thus, GILZ was identified among the most induced genes in Mo-DCs treated by Mitomycine C (75) and the Ca^2+^-targeting drug rapamycine (11) (Figure 2). Altogether, these data identify GILZ as a common intracellular marker upregulated upon treatment with several immunosuppressive therapeutic molecules. However, its importance in the drug’s effect on DCs function has been proven only for GCs so far.

## Functional Consequences of Gilz Expression in DCs

Seminal work from our laboratory established that GILZ was required for Dex-, IL-10- and TGF-β-induced downregulation of CD80, CD86, and CD83 costimulatory molecules and the increase of immunoglobulin-like transcript 3, programmed death ligand 1 (PD-L1), and IL-10 in human DCs. These results were obtained in Mo-DCs and CD34^+^ cells-derived DCs, using both lentiviral transduction to overexpress GILZ and RNA-interference on Dex-treated cells to demonstrate GILZ requirement in the tolerogenic polarization of DCs (20, 25). Other groups established that GILZ was necessary for Dex-induced CD86 downregulation in BMDCs (26), PD-L1 induction, and IL-12 inhibition in clinical grade human DCs matured with a standard cocktail (24) and IL-10 secretion by splenic DCs isolated from HGF-treated mice in the course of experimental autoimmune encephalitis (22). GILZ overexpression was then further associated with regulatory phenotype in both human and mouse DCs (11, 23, 68). Of note, in certain studies, the GILZ^hi^PD-L1^hi^ DCs retained high expression of costimulatory molecules (22, 24). Using mice constitutively overexpressing GILZ in DCs (CD11c-GILZ^hi^) and in line with the report of Cohen et al. (25), we could establish that the sole increase of GILZ levels did not alter DCs steady-state phenotype nor cytokine secretion, suggesting that the consequences of GILZ overexpression may depend on the context of GILZ induction. However, upon cognate interaction with T cells *in vivo*, GILZ^hi^ DCs produced higher amounts of IL-10 than control DCs and presented reduced MHC class II molecules levels (23), confirming their regulatory commitment. Additional insights into the importance of GILZ expression for DCs function came from total (63) and conditional (27) GILZ^KO^ mouse models. While these studies pointed to minor impact of GILZ deletion on DCs phenotype (27, 63), the cytokine response to TLR4 and TLR7 stimulations was markedly increased in GILZ^KO^ BMDCs, with higher IL-1α and β, IL-6, and IL-23 secretion (63), in line with GILZ inhibition promoting IL-12, TNF-α, and CCL5 production by Mo-DCs (20, 24, 45). In conclusion, GILZ expression in DCs promotes immature phenotype and IL-10 production while limiting secretion of Th1- and Th17-inducing cytokines (Figure 2), thereby favoring DCs polarization toward a tolerogenic profile (20–27).

The consequences of GILZ levels in DCs on their capacity to activate T-cell responses were first investigated in recall responses toward vaccine and cytomegalovirus antigens (20, 24). In these studies, Dex-induced GILZ^hi^ human Mo-DCs promoted poor autologous CD4^+^ T-cell proliferation and IFN-γ production, and this was reversed upon GILZ silencing. Along this line, clinical grade activated Mo-DCs were later shown to activate more efficiently CD8^+^ T-cell secondary responses when GILZ was knocked-down (24). Similar results were obtained for primary T-cell responses *in vivo*, the adoptive transfer of GILZ^hi^ DCs inducing poor effector T-cell (Teff) activation and IFN-γ secretion (23) while that of GILZ^KO^ (22) or GILZ-silenced (26) DCs led to enhanced CD4^+^ T cell proliferation, activation, as well as IFN-γ and IL-17 production (Figure 2). Remarkably, the poor Teff activation by GILZ^hi^ DCs was associated with the expansion of antigen-specific IL-10^+^CD4^+^ T cells in both human and mouse (20–23), which was abolished by GILZ silencing or genetic deletion in DCs (20–22). These IL-10^+^CD4^+^ T cells included Foxp3^−^ cells, a phenotype consistent with Tr1 cells (20, 21), and Foxp3^+^ Treg (20–23), and inhibited Mo-DCs-induced autologous CD4^+^ and CD8^+^ T-cell proliferation through an IL-10-dependent antigen-specific mechanism (20, 21). We further established *in vivo* that Treg expansion by GILZ^hi^ DCs was abrogated upon depletion of pre-existing Treg (20, 23), supporting the conclusion that GILZ^hi^ DCs could expand thymus-derived Treg (tTreg) more efficiently than control DCs. Such an expansion might explain the spontaneous accumulation of Treg in CD11c-GILZ^hi^ mice, with an increased frequency of inducible co-stimulator (ICOS)^hi^ Treg. Importantly, in the absence of transferred tTreg, GILZ^hi^ DCs still poorly induced conventional T cells activation, thus suggesting that GILZ^hi^ DCs were intrinsically inefficient in Teff priming, a characteristic of tolerogenic DCs (10). Altogether, the results from these studies identify GILZ expression levels in DCs as a critical switch controlling their ability to prime Teff versus Treg, mediating Dex- and HGF-induced effects on DCs phenotype, cytokine secretion, and T-cell priming capacities and required for their beneficial effects in respiratory allergic patients (21) and experimental autoimmune encephalitis (22).

Finally, GILZ expression in murine DCs has also been shown to control their antigen capture capacity. The initial report was made by Lebson et al., who observed that GILZ silencing led to increased ovalbumine (OVA) capture in BMDCs (26). Consistently, analysis of antigen uptake by DCs from CD11c-GILZ^KO^ mice revealed an increase in Dextran-FITC internalization by the splenic cDC1 subset *in vivo*. Using GILZ^KO^ and GILZ^hi^ BMDCs, we showed that GILZ selectively regulated Dextran, OVA, and Lucifer Yellow macropinocytosis, but not the phagocytosis of particulate zymosan. This control operated in immature and recently activated BMDCs, through a mechanism that may at least partly depend on the control of p38 mitogen-activated protein kinase phosphorylation (27). The fact that GILZ limits antigen internalization while Dex-treated DCs display enhanced antigen capture (76, 77) points to opposite effects of GILZ and GCs on certain DCs functions. Unexpectedly, the higher OVA macropinocytosis in GILZ^KO^ DCs did not result in increased peptide presentation to a CD4^+^ T-cell hybridoma and was even associated to reduced cross-presentation to a CD8^+^ T-cell hybridoma, despite efficient antigen degradation (27). These results suggested that the fine-tuning of antigen capture by GILZ in DCs might serve to regulate the quality rather that quantity of antigen available for presentation to T cells. It might also regulate antigen internalization for later transfer to B cells (78). Alternatively, GILZ might control additional steps in the antigen degradation and/or cross-presenting machinery of DCs. Further studies will be needed to address the physiological relevance of GILZ-mediated macropinocytosis modulation in DC subsets in adequate models *in vivo*. The link established between the macropinocytic process and the regulation of DC migration (79–81) suggests that GILZ expression might also control DCs trafficking *in vivo*, a question deserving dedicated analysis.

## Conclusion

Glucocorticoid-induced leucine zipper has initially been identified in DCs as a key mediator of GCs’ effects, piloting DCs commitment toward a tolerogenic profile. Indeed, GILZ promotes immature phenotype and IL-10 production in DCs while limiting secretion of IL-12 and IL-23, thus favoring regulatory responses. In addition, GILZ also regulates DCs access to antigen by modulating macropinocytosis, thus pointing to its regulatory role at many steps of DCs function. Recent studies have revealed that GILZ expression in DCs is constitutive and can be modulated by their ontogeny and microenvironment, with cell-specific regulatory systems. Thus, GILZ may control DCs access to antigen and the issue of their interactions with T cells in the steady state as well as in certain pathologies. Furthermore, considering the wide effects reported for GILZ in other cell types, GILZ might impact DC biology in an even broader scale with possible effects on proliferation, cell survival, and migration. Deciphering the regulation of GILZ expression in DCs as well as its role in DC subsets function *in vivo* will deserve additional studies, which will provide insights into the fine mechanisms controlling DCs and might open new avenues for therapeutic approaches.

## Author Contributions

MV and GS-L jointly wrote the review and prepared the figures.

## Conflict of Interest Statement

The authors declare that the research was conducted in the absence of any commercial or financial relationships that could be construed as a potential conflict of interest.
